# Organ-Specific Strategies in Bioprinting: Addressing Translational Challenges in the Heart, Liver, Kidney, and Pancreas

**DOI:** 10.3390/jfb16100356

**Published:** 2025-09-23

**Authors:** Mohamad Al Qassab, Moustafa Merheb, Safaa Sayadi, Pia Salloum, Zeina Dabbousi, Anthony Bayeh, Frederic Harb, Sami Azar, Hilda E. Ghadieh

**Affiliations:** 1 Department of Biomedical Sciences, Faculty of Medicine and Medical Sciences, University of Balamand, Kalhat, Al Koura, Tripoli P.O. Box 100, Lebanon; 2 Department of Medicine, Faculty of Medicine, Caucasus International University, 0141 Tbilisi, Georgia

**Keywords:** organ bioprinting, tissue engineering, bioinks, extrusion-based printing, laser-assisted bioprinting, regenerative medicine

## Abstract

Organ bioprinting is a rapidly evolving field designed to address the persistent shortage of donor organs by engineering patient-specific tissues that replicate the function and structure of natural organs. Despite significant technological advancements, bioprinting still faces major obstacles, including tissue rejection, inadequate vascularization, limited physiological functionality, and various ethical and translational challenges. In this review, we assess current bioprinting modalities, particularly extrusion-based printing, inkjet printing, laser-assisted bioprinting (LAB), and stereolithography/digital light processing (SLA/DLP), highlighting their individual strengths and limitations. We also explore different bioink formulations, focusing especially on hybrid bioinks as promising solutions to traditional bioink constraints. Additionally, this article thoroughly evaluates bioprinting strategies for four major organs: heart, liver, kidney, and pancreas. Each organ presents unique anatomical and physiological complexities, from cardiomyocyte immaturity and electromechanical mismatch in cardiac tissues to vascularization and zonation challenges in liver structures, intricate nephron patterning in kidney constructs, and immune rejection issues in pancreatic islet transplantation. Regulatory and ethical considerations critical for clinical translation are also addressed. By systematically analyzing these aspects, this review clarifies current gaps, emerging solutions, and future directions, providing a comprehensive perspective on advancing organ bioprinting toward clinical application.

## 1. Introduction

Organ failure represents an escalating global health crisis, with millions of patients worldwide dependent on transplantation for survival. However, the gap between organ demand and donor supply remains unbridgeable. For instance, in the United States alone, over 100,000 individuals await organ transplantation, with thousands dying each year before a suitable organ becomes available [1]. This shortage is further compounded by the inherent risks of transplantation, including surgical complications, lifelong immunosuppression, and limited graft longevity.

In response to these limitations, 3D bioprinting has emerged as a revolutionary solution in regenerative medicine. Over the past decade, the field has seen remarkable progress in print resolution, bioink development, and multimaterial deposition strategies [2,3,4,5,6]. This technology enables the precise layer-by-layer deposition of bioinks, comprising living cells, biomaterials, and signaling molecules, to engineer structures that mimic native tissue architecture and function with remarkable accuracy [7,8,9,10]. Importantly, this approach allows for the spatial arrangement of diverse cell types within complex geometries, paving the way for autologous, patient-specific organs that could circumvent many immunologic and logistical hurdles inherent to donor transplantation [1].

Despite the enthusiasm surrounding bioprinting, its translation from laboratory prototype to clinical-grade therapy remains fraught with scientific and regulatory challenges. These limitations stem from a set of persistent hurdles that continue to impede clinical translation, including identifying a suitable cellular source—ideally autologous cells—along with difficulties associated with harvesting, expanding, and mass producing these cells within a clinically feasible timeframe. Although advances in print fidelity, resolution, and bioink formulation have been significant, these improvements do not necessarily guarantee functional success. Many bioprinted tissues despite their structural resemblance to native counterparts fail to achieve adequate vascularization, maintain physiological activity, or integrate seamlessly with host tissues after implantation [11,12]. These challenges persist because the current bioprinting techniques struggle to recreate intricate vascular networks critical for nutrient delivery and cellular maturation and often lack the necessary mechanical and biochemical cues to support cellular function [13,14,15]. To address these limitations, scientists are creating pre-vascularized tissue structures, applying growth factors such as VEGF and integrating endothelial cells into vascular systems, stimulating the body’s natural environment with bioreactors, and using patient-specific cells and biocompatible materials to improve function and integration. Such efforts underscore the need for solutions that extend beyond structural requirements for long-term survival and functional viability.

This review explores the current landscape of organ-specific bioprinting, focusing on four complex organ systems: the heart, liver, kidney, and pancreas. Each organ presents unique anatomical and physiological demands that challenge existing bioprinting modalities.

Each system is assessed according to its modality, compatibility, cellular sources, biomaterial constraints, integration challenges, and translational barriers. The aim of this article is to evaluate the biological, technological, and regulatory landscape of organ-specific 3D bioprinting, identify the major obstacles to clinical translation, discuss ethical and regulatory considerations, and outline future directions for translating this technology into viable clinical applications. Distinct from prior reviews that primarily emphasize technical or focus on individual organs, this review offers a cross-disciplinary perspective that uniquely embeds bioprinting modalities with an in-depth evaluation of biological integration challenges and regulatory frameworks. We highlight critical gaps and future directions to provide a holistic perspective on the translational hurdles facing organ-specific 3D bioprinting.

Additionally, this article addresses often overlooked yet critical issues such as immune rejection, fibrosis, structural instability, and limited in vivo maturation. Overcoming these challenges requires coordinated scientific innovation, technological refinement, and regulatory adaptation. To this end, the global regulatory landscape for bioprinting, emphasizing inconsistencies in classification systems, approval pathways, and functional performance standards, is discussed, all of which must be harmonized to enable safe and effective clinical adoption.

We selected the heart, liver, kidney, and pancreas based on their high clinical demand and the unique anatomical and functional challenges they pose for bioprinting. Over the past decade, heart transplants have increased by 80%, liver transplants have increased by 65%, and kidney transplants have increased by 59% globally [16,17]. In the U.S., transplants reached a record 48,149 in 2024, including 27,759 kidneys, 11,458 livers, and 4572 hearts [16]. Pancreas transplantation, especially simultaneous pancreas-kidney (SPK) procedures, also continues to expand, accounting for nearly 80% of pancreas-related listings in 2023 [17]. These organs thus reflect not only high therapeutic need but also present distinct bioprinting challenges, ranging from vascular architecture and electromechanical integrity to endocrine responsiveness.

Ultimately, advancing the field of organ bioprinting depends not just on the architectural precision of laboratory constructs, but on their durability, integration, and functional performance in vivo. Despite impressive fabrication advances, clinical translation remains limited: challenges surrounding construct longevity, biomechanical fidelity, and reproducible manufacturing standards continue to impede therapeutic deployment [18]. Indeed, as Sun et al. note, only a handful of constructs demonstrate sufficient maturity and integration to warrant further translational development [19].

## 2. Bioprinting Modalities and Materials: Capabilities, Limitations, and Strategic Alignment

To systematically compare the current state of bioprinting strategies across different organ systems, we focus on four organs: the heart, liver, kidney, and pancreas. These were selected based on their high clinical demand, frequency of transplantation needs, and distinct engineering challenges. Each organ presents unique anatomical and physiological characteristics that influence bioprinting strategy design, from microarchitecture and vascular density to functional cell types and integration requirements.

The classification of anatomical complexity into high, moderate, and low follows an established conceptual framework in tissue engineering, which stratifies organs based on the intricacy of their structure and function. Solid organs such as the liver and kidney are considered highly complex due to their densely packed, multi-zonal architecture, metabolic heterogeneity, and high perfusion requirements. The heart, while also structurally sophisticated, is classified as moderately complex owing to its anisotropic yet rhythmically patterned architecture. In contrast, organs such as the pancreas are categorized under lower complexity because of their relatively simpler microstructural hierarchy and functional compartments, despite the clinical importance of achieving β-cell maturation and immune protection. This classification rationale aligns with prevailing views in the organ engineering field.

To contextualize the specific bioprinting challenges and advances across different organs, Table 1 presents a comparative summary of key hurdles, current strategies, remaining gaps, and future directions for the heart, liver, kidney, and pancreas. Organs were categorized into high, moderate, or low complexity based on their structural organization, cellular heterogeneity, vascularization requirements, and functional integration.

As outlined in Table 1, each organ system poses distinct demands that require tailored bioprinting approaches. For example, while the heart faces issues such as immature electromechanical behavior and poor integration, the liver’s primary hurdles include replicating metabolic zonation and achieving sufficient perfusion. These differences underscore the necessity of organ-specific design principles, which are further unpacked in the following sections.

### 2.1. Overview of Bioprinting Modalities

Bioprinting comprises a diverse array of fabrication techniques, each characterized by unique mechanisms of bioink deposition, resolution thresholds, and material compatibility. Rather than constituting a single, unified method, bioprinting technologies diverge significantly in terms of their operational parameters and suitability for engineering specific tissue types. The four principal modalities employed in contemporary bioprinting research are extrusion-based printing, inkjet printing, laser-assisted bioprinting (LAB), and stereolithography or digital light processing (SLA/DLP) [25,26].

Extrusion-based bioprinting is currently the most widely adopted modality due to its operational simplicity and compatibility with a broad range of bioink viscosities [27]. In this technique, bioinks are extruded through a mechanical or pneumatic nozzle, enabling the fabrication of large, cell-dense constructs. Bulk tissue engineering refers to the fabrication of three-dimensional, volumetric tissue constructs with clinically relevant thickness, which require vascularization and nutrient supply strategies to maintain cell viability and functionality. This approach is particularly well-suited for bulk tissue engineering applications, such as the fabrication of blood vessels and cardiac tissue constructs, as demonstrated by Forgacs and colleagues. Moreover, Yan et al. implemented extrusion-based bioprinting for the design of bone tissue scaffolds using a multi-nozzle deposition system while Ouyang et al. formed 3D cell-laden structures made of embryonic stem cells [28,29]. The process may impose considerable shear stress on encapsulated cells during extrusion, which may compromise cellular viability [30]. However, when controlling certain parameters such as cell density, bioink used and the duration for bioprinting large constructs, cellular viability is better maintained. Additionally, extrusion systems offer limited spatial resolution, generally in the range of 100 to 300 μm, making them less effective for applications requiring fine structural detail [7,8,31].

Inkjet bioprinting, by contrast, utilizes thermal or piezoelectric actuation to eject precise droplets of bioink onto a substrate. This technique enables higher spatial resolution than extrusion methods and is advantageous for patterning multiple cell types or bioactive molecules in controlled gradients. This was applied in the development of a 3D liver-on-a-chip consisting of endothelial cells and hepatocytes, which make up the different layers of the liver [32]. It was also used to fabricate cardiac tissue as reported by Xu et al., who presented a cost-effective tool [33]. As for bone bioprinting, collagen-calcium phosphate (col-CaP) scaffolds were fabricated by Inzana et al. using type I collagen and phosphoric acid (H_3_PO_4_) [34]. Another use of inkjet bioprinting is that of cancer research whereby Demerci and group fabricated ovarian cancer tumor tissue and a normal human fibroblast cell line to further understand the relation between tumor and stromal cells [35]. Despite these advantages, inkjet bioprinting is restricted to low-viscosity bioinks, limiting its applicability to constructs that require mechanical integrity. Furthermore, nozzle clogging and inconsistent droplet formation can hinder the reproducibility of printed tissues [36].

Laser-assisted bioprinting represents a nozzle-free alternative that leverages laser-induced forward transfer (LIFT) to propel discrete volumes of bioink from a donor ribbon to a receiving substrate. It offers precise and rapidly formed patterns of bioprinting [37]. This approach offers excellent spatial precision and circumvents common issues associated with nozzle obstruction, such as viscosity [38]. It also supports a wide range of cell densities and bioink formulations while maintaining high cell viability, often exceeding 90%. Wu et al. made use of laser-assisted printing technology by constructing human umbilical vein endothelial cells (HUVECs) adding to it an extra layer of human umbilical vein smooth muscle cells (HUVSMCs) to ensure longer survival of the blood vessel bioprinted [39]. As for neurological disorders, conductive hydrogel was fabricated using laser-assisted bioprinting to aid in the recording of biological signals and stimulating living tissues [40]. Moreover, stem cell graft generation is possible via this method, entailing no damage to the cells [41]. Nevertheless, the high cost, technical complexity, and limited scalability of laser-assisted systems have restricted their widespread adoption, particularly for clinical-scale tissue fabrication [42].

Stereolithography and digital light processing, categorized as photopolymerization-based techniques, achieve superior resolution, often below 50 μm, by using light to selectively cure photocrosslinkable bioinks layer-by-layer. These techniques are highly effective in replicating the intricate microarchitectures of native tissues such as hepatic sinusoids or renal tubules [43]. However, their use is constrained by the need for low-viscosity, photosensitive materials, which may limit the diversity of available bioinks. Moreover, exposure to ultraviolet or visible light can pose cytotoxic risks to encapsulated cells, thereby necessitating careful optimization of photoinitiator concentrations and exposure parameters to preserve cell viability [3,27].

A comparative overview of key bioprinting modalities—including resolution, bioink viscosity compatibility, cell viability, and their respective strengths and limitations—is provided in Table 2.

While traditional bioprinting techniques, such as extrusion, inkjet, and stereolithographic methods, remain cornerstones of construct fabrication, emerging approaches like volumetric bioprinting (VBP) offer a compelling alternative. VBP enables rapid layer-free production of complex, cell-laden geometries on the order of centimeters in mere seconds, potentially overcoming speed and scalability limitations of conventional modalities [44,45]. Early demonstrations in bone-like tissue constructs and multi-material platforms highlight VBP’s applicability to biologically relevant architectures [46,47]. While not yet incorporated into organ-scale models discussed here, volumetric strategies represent a promising avenue for future bioprinting innovations.

### 2.2. Bioink Formulation: Trade-Offs Between Biofunctionality and Printability

The bioink serves as the fundamental building block in bioprinting, integrating structural, biological, and functional roles. It provides not only the physical matrix necessary for shaping tissue architectures but also the microenvironmental cues that influence cell viability, proliferation, differentiation, and eventual tissue maturation. For bioprinting to succeed, bioinks must simultaneously meet mechanical and rheological demands for printability while supporting the biological requirements for tissue development and in vivo functionality [48,49,50]. However, this dual mandate presents an inherent challenge; bioinks with ideal mechanical fidelity often lack the biochemical complexity needed to support tissue-specific cellular functions, whereas biologically rich matrices typically suffer from poor printability, structural instability, or batch-to-batch inconsistency [4,51,52,53].

Additionally, it is important to note that bioink requirements can vary significantly based on the bioprinting technique employed. For example, extrusion-based printing demands bioinks with specific shear-thinning properties and mechanical robustness to maintain shape fidelity during deposition, whereas inkjet bioprinting favors low-viscosity bio-inks to enable droplet formation and precise placement. Similarly, laser-assisted printing requires bioinks with particular optical and thermal properties to ensure viability and print accuracy [54,55]. These methods depend on variations, further complicating bioink design and necessitating tailored formulations for each printing modality.

Bioinks are typically composed of natural polymers, synthetic polymers, or hybrid combinations thereof. Natural polymers such as collagen, alginate, gelatin, hyaluronic acid, fibrin, and decellularized extracellular matrix (dECM) are commonly used due to their inherent biocompatibility and ability to mimic the native extracellular matrix (ECM) [48,56]. These materials provide favorable microenvironments for cell adhesion, proliferation, and differentiation, especially in tissues like skin, cartilage, and liver. However, many of these natural hydrogels lack sufficient mechanical integrity, degrading rapidly under physiological conditions or collapsing during post-printing handling. Alginate, for example, is widely used due to its ease of ionic crosslinking and biocompatibility, yet it lacks intrinsic cell adhesion motifs and typically requires blending with gelatin or RGD peptides to support cellular function [48,57].

In contrast, synthetic bioinks such as polyethylene glycol (PEG), polycaprolactone (PCL), Pluronic F127, and polyvinyl alcohol (PVA) offer tunable mechanical strength, degradation rates, and chemical functionality [4,58,59]. These polymers allow precise control over rheological behavior and are often used for load-bearing constructs or where shape fidelity is critical [60]. For example, PCL has been used extensively in bone and vascular tissue bioprinting due to its strength and slow degradation profile [61]. However, these materials are generally bioinert and require functionalization, such as incorporation of peptide motifs like RGD or growth factors, to promote cellular responses [62,63].

To reconcile the limitations of both natural and synthetic systems, hybrid bioinks have gained prominence [55]. These blends aim to integrate the printability and structural stability of synthetic polymers with the bioactivity of natural components [64]. For instance, GelMA (gelatin methacryloyl) crosslinked with PEGDA (polyethylene glycol diacrylate) forms a photopolymerizable network with both biocompatibility and shape fidelity [4,48,65]. Similarly, alginate-PCL and alginate-nanocellulose composites have demonstrated improved mechanical and biological performance in meniscal and adipose tissue engineering [66,67,68]. In a study by Semba et al., an alginate–gelatin–nanocellulose bioink exhibited favorable rheology, high print fidelity, and supported cell viability for meniscal reconstruction, showcasing the potential of multi-component formulations [66].

Another innovation in bioink design involves the use of dECM bioinks derived from specific tissues [69,70]. These materials retain tissue-specific biochemical cues, including collagen IV, laminin, fibronectin, and glycosaminoglycans, which can support stem cell differentiation and enhance tissue-specific functions [5,49,71]. For instance, liver-derived dECM has been shown to improve albumin and urea production in hepatocyte-laden constructs, while pancreas-specific dECM enhances β-cell maturation and insulin expression [23,52]. However, these dECM bioinks suffer from batch-to-batch variability, low mechanical strength, and potential immunogenicity due to residual cellular material. Furthermore, their derivation from animal tissues introduces regulatory and ethical concerns, especially with regard to zoonotic pathogen transmission and GMP compliance [51].

Supportive materials like gelatin microparticle baths used in FRESH (Freeform Reversible Embedding of Suspended Hydrogels) printing represent an alternative approach to improving the printability of soft hydrogels. The FRESH technique enables the deposition of soft, bioactive bioinks within a temporary support matrix, allowing complex structures to be fabricated without gravitational collapse. Bliley et al. demonstrated the use of FRESH to bioprint human cardiac tissue that exhibited spontaneous rhythmic contractions and mechanical integrity in vitro [20,72]. Nevertheless, FRESH requires careful optimization of post-print support bath removal and is not yet scalable for large or vascularized tissues [20,48].

To contextualize recent progress in bioprinting across organ systems, Table 3 summarizes landmark studies in heart, liver, kidney, and pancreas bioprinting. The table highlights the cell sources, bioinks, bioprinting strategies, and key functional outcomes of each study, offering a comparative view of translational readiness and technological maturity across organ platforms.

Table 4 summarizes several leading bioink components and hybrid formulations along with their applications, advantages, and limitations.

### 2.3. Beyond Printability: Designing for Function and Integration

Early bioprinted constructs were often evaluated by their print accuracy, structural stability, and resemblance to native tissues. While important for initial development, those form-based benchmarks do not reflect clinical potential. High-resolution constructs that lack tissue-specific function, vascularization, or host integration remain non-therapeutic. Moving forward, success must be defined by functional performance and how well the construct mimics real organ behavior and integrates into the body [11,92].

This shift is particularly important for organ-level constructs. For example, a bioprinted heart patch must do more than visually resemble myocardium; it must beat synchronously, respond to electrophysiological cues, and endure cyclic mechanical stress over time, and not only a visual resemblance. Similarly, a hepatic construct should have key functions such as producing albumin, urea, and enzymes in correct spatial patterns, rather than just containing liver-like cells. However, many preclinical studies still rely on static histology or short-term cell survival tests, overlooking critical functional assessments [21,63].

A major challenge in designing functional bioprinted tissues is achieving effective vascularization. Without a perfusable microvascular network, tissues quickly develop hypoxia, leading to cell death and loss of function. Partial solutions include sacrificial templating to create perfusable channels, coaxial extrusion of endothelial lumens, and integration with self-organizing organoids, which can initiate spontaneous vasculature under controlled culture conditions [3,27,93].

These approaches have demonstrated improved outcomes-for example, coaxially printed hydrogel cores have supported functional β-cell survival and insulin response [23], while hollow microchannel geometries in hepatic and renal constructs enhanced nutrient diffusion and viability gradients [22,51], in addition miniaturized organoids of the heart, liver, and lung can spontaneously develop their own vascular networks under defined culture conditions [94].

Integration of these constructs with host vasculature remains unpredictable and poorly characterized. Successful anastomosis has mostly been reported in small animals over short periods, with few studies using quantitative methods like Doppler imaging or micro-CT to measure blood flow [21]. The lack of standardized in vivo tests also hinders comparison across studies. As noted by Ricci et al., regulatory progress is hampered not by a lack of innovation, but by the absence of validated assays that measure what truly matters: functional tissue integration, systemic benefit, and long-term safety [12].

A major challenge in bioprinting functional organs is managing the immune response to constructs. Even with autologous or immunomodulated cells, residual crosslinkers, scaffold materials, or incomplete decellularization can trigger chronic inflammation or fibrosis, especially when using animal-derived dECM as reported by Osidak et al. [49]. This is critical for liver and kidney bioprinting, where fibrotic encapsulation impairs perfusion and organ function. Current studies typically assess host responses only up to 28 days, which is insufficient to predict long-term rejection or fibrosis [11,12].

A key limitation in the field is the lack of standardized, quantitative definitions of “function” in bioprinted tissues. Most studies rely on qualitative markers-such as viability or hormone secretion- without benchmarking against native performance. For true clinical relevance, function must be organ-specific, measurable, and sustained over time.

### 2.4. Gaps and Future Directions in Bioprinting Modalities

Despite significant strides in 3D bioprinting over the past decade, the field continues to grapple with a number of critical limitations that prevent the realization of clinically viable, fully functional organ constructs. While proof-of-concept studies have demonstrated that basic tissue architecture and even limited organ-specific functions can be replicated in vitro, the transition toward scalable, vascularized, immunocompatible, and functionally mature constructs remains elusive [42,92]. The bottlenecks lie not only in technological capability but also in conceptual design, biological modeling, and system-level integration.

A key research gap centers on the lack of modality capable of meeting all design and functional requirements for specific organ systems. For instance, extrusion-based printing is widely adopted for its versatility and compatibility with viscous, cell-laden bioinks, but it falls to generate high-resolution microstructures essential for tissues such as the kidney or liver, where capillary-level perfusion and complex tubular geometries are essential for organ-level function [3,22,95,96]. Conversely, SLA and DLP printing offer superior resolution but are restricted by the need for photopolymerizable bioinks and by light-induced cytotoxicity that may compromise long-term viability of encapsulated cells [43]. The trade-offs inherent in each modality have led researchers to consider hybrid approaches, but these remain in the early stages of technical development and raise new challenges regarding synchronization, bioink compatibility, and inter-modality fidelity.

Moreover, vascularization remains an unsolved challenge, particularly for thick or metabolically demanding tissues where diffusion alone is insufficient to support cellular viability. Constructs intended to mimic liver lobules, renal cortex, or pancreatic islets require not only perfusable channels but also biologically integrated, hierarchically branched vascular networks to enable nutrient delivery, waste removal, and immune regulation. While one proposed strategy involves using extrusion printing to deposit bulk parenchymal tissue, sacrificial bioinks to create vascular channels, and light-based lithography for intricate structural elements such as biliary ducts or renal tubules [97], this approach remains technically limited in several key areas.

For example, the FRESH platform developed by Bliley et al. demonstrated the feasibility of printing soft tissue constructs, such as miniaturized heart ventricles within gelatin microparticle support matrices, which could be subsequently perfused [20]. Although these constructs showed promising features such as complex geometry and ejection activity, the system still falls short in areas critical for clinical application. Specifically, such hybrid approaches continue to suffer from low throughput, limited scalability, and poor integration of functional subsystems-this is significant when transitioning from small to larger scales. Moreover, these constructs lack the mechanical robustness and functional redundancy of native tissues. These limitations show the need for integrated multimodal platforms where real-time feedback and quality control are incorporated.

Beyond fabrication challenges, another significant gap is the reproducibility and standardization of bioprinted constructs. As Ricci et al. report, the field suffers from batch-to-batch variability in cell sources, bioink composition, printer calibration, and crosslinking parameters, all of which directly impact the structural and functional fidelity of printed tissues [12]. These variabilities are problematic for constructs intended for clinical manufacturing, where even minor deviations can lead to unacceptable performance or safety risks. Unlike traditional pharmaceuticals or even conventional tissue-engineered products, bioprinted constructs often involve patient-specific biological inputs, making them more difficult to validate. Compounding the problem is the lack of open-source, standardized bioprinting protocols or universally accepted benchmarks for evaluating construct performance across different laboratories [12,58]. As a result, findings are often non-replicable across laboratories; thus, the need for uniform standards in biofabrication, including reference materials, calibration protocols, functional testing assays, and cross-platform validation frameworks, is becoming more essential. These standards will also facilitate regulatory alignment, multi-center studies, and the eventual commercial scalability of bioprinted products.

Furthermore, the field continues to face a critical gap in long-term functional validation of bioprinted constructs in preclinical models. Most studies monitor construct behavior for only short durations—few days to weeks—and focus primarily on early-stage markers such as viability, morphology, and initial germ expression. However, as highlighted by Cross-Najafi et al. and Salg et al., a major research gap lies in the lack of long-term, specific functional data, such as sustained detoxification in new hepatic constructs, stable electrolyte reabsorption in renal models, and glucose-sensitive insulin secretion in engineered pancreatic tissue [11,21]. This gap significantly hinders progress toward meeting regulatory expectations for investigational human use.

A growing body of evidence highlights a critical research gap: the disconnect between the in vitro and in vivo functional behavior of cellular phenotypes. Despite appearing phenotypically appropriate, many iPSC-derived cardiomyocytes, hepatocytes, or pancreatic β-cells used in bioprinting remain immature profiles that do not translate into functional competence after implantation [23,24]. These cells often lack adult electrophysiological properties, secrete insufficient levels of essential proteins, or exhibit low responsiveness to physiological stimuli. While biophysical conditioning, like mechanical strain, electrical pacing, and co-culture systems, has been proposed to enhance maturation, their integration into bioprinting workflows remains underdeveloped and poorly standardized [58,63]. Furthermore, there is a lack of standardized protocols to evaluate and compare maturation outcomes across platforms, leaving a significant gap in optimizing cell functionality for clinical applications.

Immune compatibility remains a major, underexplained barrier in bioprinting research. As Osidak et al. and Ribezzi et al. reported, even when autologous or low-immunogenicity cells are used, residual scaffold materials, incomplete decellularization, or inflammatory bioink components can trigger host responses ranging from mild inflammation to fibrotic encapsulation [49,52]. These immune reactions-ranging from mild inflammation to fibrotic encapsulation- highlight a critical research gap: the field lacks reliable, long-term models to study immune-biomaterial interactions under chronic implantation conditions. This is especially problematic for immune-sensitive organs such as the liver and pancreas, where heightened immune surveillance and the risks associated with systemic immunosuppression demand precise control over host response. Despite that, there is limited understanding of how specific biomaterial formulations, degradation products, or cell states contribute to long-term.

Despite a growing body of promising studies, the field continues to lack a coherent translational framework-a significant research gap that impedes clinical progress. There is no current standardized set of milestones, performance metrics, or validation pathways to guide bioprinted constructs from early-stage feasibility to clinical implementation. As Ricci et al. argue, one clinical missing piece is the development of modular, stepwise trial designs that prioritize partial or supportive functions, such as hepatic assist devices, nephron patches, or insulin-secreting implants, before reaching full replacement [12]. However, few studies are structured around his incremental approach, and there is limited guidance on how to define success at each stage, from integration to function and safety. This lack of translational strategy slows the accumulation of clinically actionable data and increases the risk of failure in late-stage development.

## 3. Cardiac Bioprinting—From Rhythmic Constructs to Functional Myocardial Repair

### 3.1. Anatomical and Functional Demands of the Heart

The human heart is a biomechanically and electrophysiologically complex organ, making it one of the most demanding targets for regenerative bioprinting. It is composed of multiple, functionally specialized cell types, primarily cardiomyocytes, fibroblasts, vascular endothelial cells, and smooth muscle cells, arranged in a highly anisotropic and hierarchical manner. This alignment is critical for the propagation of electrical signals and the generation of synchronized contractions. The left ventricular myocardium, for example, exhibits helically oriented fiber layers that optimize ejection fraction and mechanical efficiency. Any construct intended to replace or augment cardiac tissue must replicate not only the structural layout but also the functional integration of excitation–contraction coupling [20].

Moreover, cardiac tissue is subject to continuous mechanical stress due to the cyclic nature of the heartbeat, which includes rapid depolarization, forceful contraction, relaxation, and refilling. Unlike other organs that may tolerate partial or non-uniform activity, the heart operates under all-or-nothing physiological constraints: a lack of synchrony or mechanical integrity in even a small region can lead to conduction abnormalities or functional failure [63]. As such, cardiac bioprinting occupies the high-complexity, low-readiness quadrant in any organ–modality compatibility matrix.

**Figure 1:**
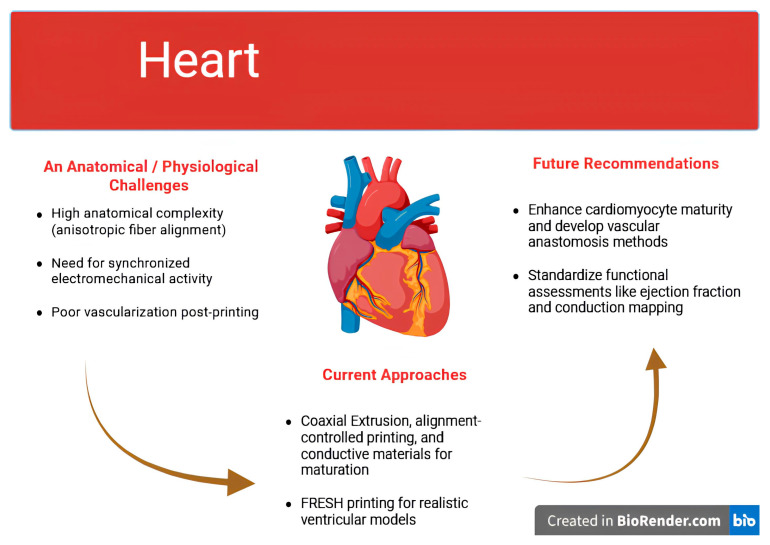
Heart Bioprinting: Engineering Approaches to Reproduce Electromechanical Function. Created with BioRender.com.

Figure 1 illustrates recent progress in 3D-bioprinted cardiac constructs. These models attempt to replicate myocardial architecture and electromechanical function, incorporating vascular channels and contractile cardiomyocytes to mimic native tissue physiology.

### 3.2. Advances in Bioprinted Cardiac Patches

To date, most cardiac bioprinting efforts have focused on the development of myocardial patches designed to be implanted over areas of infarct or fibrosis. These patches aim to support electromechanical repair by restoring contractile capacity and facilitating vascularization. Initial prototypes often employed simple hydrogel scaffolds, such as GelMA or alginate, embedded with cardiomyocytes derived from human induced pluripotent stem cells (hiPSCs) or neonatal rat hearts. While these early patches demonstrated spontaneous beating and moderate electrical propagation in vitro, they generally failed to integrate electrically with native myocardium in vivo, largely due to immature cellular profiles and lack of perfusion [63,92]. The vascularization gap seen in engineered cardiac patches may be addressed by developmental self-assembly. A 2025 study demonstrated that mini-heart organoids formed their own vascular networks, highlighting the potential of self-patterned vasculature as a complementary strategy to coaxial or sacrificial approaches [94].

Recent innovations have improved upon these limitations by leveraging coaxial extrusion and alignment-controlled printing [98]. Coaxial extrusion enables the fabrication of hollow filaments with perfusable lumens, which can be endothelialized to form primitive vascular networks. These channels allow the transport of nutrients and oxygen deep into the patch and provide a scaffold for host vessel infiltration after implantation [3]. Meanwhile, alignment-controlled bioprinting, wherein nozzle pathing is manipulated to orient cells and extracellular matrix fibers, has been shown to enhance the anisotropy of engineered cardiac tissue. Such alignment mimics native myocardial architecture and improves the velocity and directionality of electrical conduction [20].

In an exemplary study, Bliley and colleagues used the FRESH technique to bioprint a miniaturized left ventricle chamber with a tri-layered geometry and integrated vascular architecture [20]. The construct was seeded with hiPSC-derived cardiomyocytes and exhibited visible systolic and diastolic wall motion under electrical pacing [99].

The FRESH-based model described in Bliley et al. represents one of the most functionally advanced cardiac constructs to date, although it remains limited in scale, durability, and pressure-generating capacity [20,100].

### 3.3. Immaturity and Electromechanical Integration

A consistent limitation across nearly all cardiac bioprinting studies is the immaturity of the derived cardiomyocytes. While hiPSCs offer an autologous, renewable source of cardiomyocytes, the differentiated cells typically exhibit fetal-like electrophysiological and structural characteristics. These include disorganized sarcomere assembly, reduced action potential upstroke velocity, incomplete t-tubule formation, and underdeveloped calcium handling capabilities [63]. Such deficiencies reduce the strength and synchrony of contractions, and, more critically, increase the risk of arrhythmias upon implantation.

This immature phenotype poses a serious challenge when integrating with native adult myocardium. The mismatch in action potential duration, conduction velocity, and refractory periods can create zones of electrical heterogeneity, predisposing to reentrant circuits and ventricular tachyarrhythmias [20]. Although strategies such as mechanical conditioning, electrical pacing, or the incorporation of conductive materials, like gold nanowires, graphene, and carbon nanotubes, have been employed to enhance maturation, these approaches introduce new biocompatibility and long-term safety concerns [63]. However, as Harley et al. note, while conductive modifications improve synchrony in vitro, their translation is hindered by fibrosis, immune reactivity, and inconsistent performance in large-animal models, highlighting the need for safer, integrable alternatives in electromechanical coupling [96].

### 3.4. Mechanical Demands and Host Integration

Beyond electrical synchrony, engineered cardiac tissues must meet the mechanical demands of continuous cyclic loading. A construct that is too stiff can impede diastolic filling and reduce stroke volume, whereas an overly compliant patch may not contribute to systolic ejection and may mechanically decouple from the contracting myocardium. Balancing these properties requires fine-tuning of bioink stiffness, degradation kinetics, and cellular remodeling capacity [92].

Integration with the host myocardium also demands robust vascularization. Without a dense capillary network, the center of the patch becomes hypoxic and undergoes necrosis. Although coaxial and sacrificial printing techniques have yielded constructs with internal perfusion channels, their ability to establish long-term, functional anastomoses with host vasculature is not yet proven. Most studies to date report short-term survival and limited improvements in ejection fraction in small-animal models, with few extending to large animals or multi-month timeframes [3,20].

### 3.5. Functional Assessment and Clinical Readiness

A major concern in the translation of cardiac bioprinting is the lack of standardized, function-oriented validation protocols. Common metrics such as spontaneous contraction, troponin expression, or patch attachment are not adequate indicators of therapeutic benefit. Instead, clinical-grade constructs must be evaluated based on quantitative improvements in cardiac output, conduction velocity mapping, reduction in infarct size, prevention of adverse remodeling, and absence of arrhythmogenesis [12,101].

Furthermore, regulatory expectations will likely include chronic safety data in large-animal models, including assessments of immune response, fibrosis, and device–tissue mechanical integrity under physiological stress [12]. Current trials are unlikely to be approved for whole-chamber replacement in the near term. Instead, first-in-human applications may focus on adjunctive therapies, such as bioengineered epicardial patches or rhythm-stabilizing constructs, that mitigate arrhythmia risk or promote angiogenesis in ischemic regions [24].

## 4. Liver Bioprinting—Function, Zonation, and Post-Implantation Challenges

### 4.1. The Liver as a Bioprinting Target: Unique Advantages and Complexities

The liver stands out as a particularly appealing yet complex target for bioprinting due to its unique regenerative capacity, segmental anatomy, and highly zonated metabolic functions. Unlike the heart or kidney, even partial restoration of liver function can offer substantial clinical benefits, especially in cases of acute liver failure or metabolic disorders. Moreover, the liver is one of the few organs capable of regenerating large portions of lost tissue, suggesting that engineered implants could serve as both temporary and long-term solutions [21].

However, the liver’s biological and anatomical intricacies present substantial challenges. Native hepatic architecture is organized into functional units known as lobules, which are hexagonal in shape and consist of hepatocytes arranged around a central vein, with blood flowing in from portal triads at the periphery. This radial flow creates steep gradients of oxygen, hormones, and nutrients, giving rise to a phenomenon known as metabolic zonation [11]. Hepatocytes in different zones exhibit specialized functions: periportal cells, Zone 1, are dominant in oxidative metabolism and gluconeogenesis, while pericentral cells, Zone 3, are more involved in glycolysis, xenobiotic detoxification, and ammonia metabolism [21]. Failing to recreate this functional heterogeneity in bioprinted constructs often results in suboptimal or incomplete hepatic function, even if hepatocyte viability and albumin production appear intact.

Another layer of complexity arises from the liver’s dual vascular input, via the portal vein and hepatic artery, and its sinusoidal capillary networks lined with fenestrated endothelial cells. These sinusoids are not merely conduits for perfusion but also serve immunological and metabolic functions. Therefore, to create a functionally relevant hepatic tissue, bioprinted constructs must replicate both lobular geometry and microvascular architecture at a level of fidelity that supports differential oxygen delivery and intercellular signaling [11].

**Figure 2:**
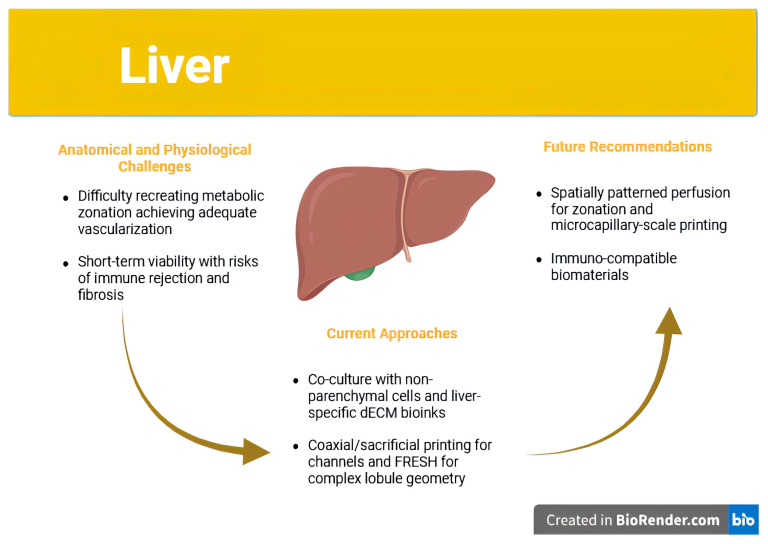
Liver Bioprinting: Vascularization, Zonation, and Immunological Considerations. Created with BioRender.com.

As shown in Figure 2, liver bioprinting efforts have advanced toward multicellular constructs that recapitulate hepatic zonation and vascular perfusion. These designs incorporate hepatocytes, endothelial cells, and supporting stromal cells arranged to mirror native lobular structure.

### 4.2. Cell Sources and Bioinks: Beyond Viability

The functional success of a bioprinted liver construct is contingent upon the quality of its cellular constituents. While primary human hepatocytes remain the gold standard due to their mature enzyme expression profiles, they are notoriously difficult to expand ex vivo and rapidly lose phenotype in standard culture conditions. In contrast, hepatocyte-like cells (HLCs) derived from hiPSCs are more scalable but often retain fetal-like features, including lower levels of cytochrome P450 enzymes and impaired bile acid metabolism [51]. These limitations significantly reduce their utility for disease modeling and drug metabolism studies.

To address this, recent strategies have employed co-culture systems that integrate hepatocytes with non-parenchymal liver cells such as liver sinusoidal endothelial cells (LSECs), Kupffer cells, and hepatic stellate cells. These multicellular constructs more closely mimic the liver’s native microenvironment and support enhanced hepatocyte function through paracrine signaling and matrix remodeling [3,11]. However, the spatial organization of these cell types is critical, and most bioprinting techniques have yet to achieve the resolution and reproducibility required for accurate placement.

Bioink composition further influences construct performance. Natural hydrogels such as gelatin, alginate, and fibrin provide a biocompatible scaffold but lack mechanical strength and degrade rapidly. Synthetic hydrogels like polyethylene glycol (PEG) offer mechanical tunability but require functionalization to support cell adhesion and differentiation [4]. One of the most promising bioink innovations for liver applications is the use of liver-specific dECM. These dECM hydrogels retain the organ’s native biochemical milieu, including collagen IV, fibronectin, and glycosaminoglycans, and have been shown to enhance albumin production and urea synthesis in embedded hepatocytes [49,52,102]. Nevertheless, they also carry risks of immunogenicity, batch variability, and poor structural integrity, limiting their scalability and regulatory acceptance [51].

FRESH bioprinting has emerged as a novel technique to address structural limitations by enabling the printing of soft bioinks within a gelatin microparticle bath that supports complex 3D shapes during printing. Constructs printed using FRESH have achieved functional geometries with embedded vasculature and enhanced hepatic activity, but the post-processing steps required to remove the support material and ensure perfusion present additional hurdles [20,72].

### 4.3. Vascularization and Zonation: Central Barriers to Functional Equivalence

One of the foremost challenges in liver bioprinting is achieving functional vascularization. The liver receives approximately 25% of the cardiac output and relies on a low-pressure, high-volume system to perfuse sinusoids and deliver metabolic substrates. Without sufficient perfusion, even the most sophisticated cell-laden constructs are prone to central necrosis and functional decline. Coaxial bioprinting, which enables the formation of hollow, perfusable channels surrounded by supportive matrix and endothelial cells, has shown promise in creating hepatic lobule-like geometries [3,97]. Other approaches using sacrificial inks, such as Pluronic F127, can form networks of microchannels that are endothelialized post-printing to simulate sinusoidal flow [97].

However, while these strategies may prevent hypoxia and improve short-term viability, they have not yet recapitulated the full complexity of hepatic blood flow or fenestrated capillary exchange. Moreover, perfusion alone is not sufficient: it must be spatially patterned to recreate metabolic zonation, which remains largely unachieved in existing constructs. As Cross-Najafi et al. argue, this failure to recreate functional gradients impairs liver-specific processes such as ammonia detoxification and lipid metabolism, even in morphologically correct tissues [21].

Another issue is the inadequate resolution of most bioprinting systems for generating microcapillaries with diameters below 20 μm, which are necessary for effective exchange between blood and hepatocytes. While light-based techniques like DLP could theoretically achieve such resolution, their limitations in bioink compatibility and light toxicity have prevented widespread adoption in liver applications [43].

### 4.4. Immune Rejection and Fibrotic Encapsulation

Even when functional perfusion and hepatocyte activity are achieved, post-implantation survival remains a persistent obstacle. Bioprinted hepatic tissues are frequently subject to immune rejection and fibrotic encapsulation, both of which diminish their clinical utility. For example, studies using xenogeneic or allogeneic dECM have reported significant macrophage infiltration, fibrous capsule formation, and eventual construct degradation [49,51]. These responses are often triggered by residual DNA fragments, poorly crosslinked scaffolds, or synthetic polymer degradation products.

Despite the clinical relevance of this issue, most published studies assess host response for no more than 14–28 days, providing an incomplete picture of chronic inflammation, fibrosis, or immunological tolerance. As noted by Ricci et al., the lack of longitudinal studies and absence of standardized immune assessment tools make it difficult to compare the biocompatibility of constructs across different platforms or guide regulatory approval processes [12]. Furthermore, immunosuppressive regimens used in small-animal studies may not be feasible in human trials, necessitating the development of immuno-silent or immuno-modulatory biomaterials.

### 4.5. Toward Functional Trials: Realistic Clinical Applications

Given the formidable challenges of whole-organ replacement, the initial clinical applications of liver bioprinting are likely to be partial or supportive in nature. Constructs designed for metabolic assistance, such as detoxification patches or implantable enzyme-producing units, may offer therapeutic benefit without the need for complete hepatic architecture or full vascularization [21]. These constructs could be employed as bridge-to-transplant therapies in acute liver failure or as adjuvants in patients with inborn errors of metabolism. They would require lower functional thresholds compared to full organ replacements and would therefore be more feasible under current technological and regulatory constraints. Clinical validation of these constructs will depend on organ-specific outcome measures. Key metrics may include reductions in serum ammonia, bilirubin, or transaminase levels, maintenance of synthetic function and integration with host vasculature as assessed by contrast-enhanced imaging. These endpoints should be evaluated over several months in large-animal models to ensure sustained function and biocompatibility prior to human trials [12].

## 5. Kidney Bioprinting—Navigating Anatomical Complexity and Translational Realities

### 5.1. The Kidney as the Most Technically Challenging Organ for Bioprinting

Among all bioprinting targets, the kidney presents the most daunting biological and engineering challenges. This organ is responsible for a suite of tightly regulated functions, including blood filtration, fluid and electrolyte balance, pH regulation, endocrine activities, renin and erythropoietin secretion, that rely on the intricate coordination of over a million nephrons per kidney. Each nephron includes a highly specialized glomerulus for filtration, followed by a series of tubular segments that reabsorb, secrete, and concentrate solutes in a sequential and tightly zoned manner [22].

Bioprinting a functional nephron is not merely a matter of structural mimicry; it demands recapitulation of multiple, sequential physiological processes, each dependent on unique cellular phenotypes and microarchitectures. For instance, the proximal tubule performs bulk reabsorption of water and solutes, while the loop of Henle establishes countercurrent gradients essential for urine concentration. The distal tubule and collecting duct respond to hormonal signals such as aldosterone and vasopressin to regulate final solute composition. These processes require not only spatially patterned epithelium but also a perfusable vascular network capable of selective exchange, mechanical support, and dynamic responsiveness to systemic stimuli [3].

Moreover, the kidney’s development is driven by reciprocal interactions between the ureteric bud and the metanephric mesenchyme, two distinct embryonic lineages. Replicating this branching morphogenesis in vitro, let alone via bioprinting, has yet to be achieved with any degree of fidelity. While other organs, such as the liver or pancreas, can demonstrate partial function in simplified models, the kidney exhibits a threshold effect: unless multiple nephron segments operate in tandem and communicate with the vasculature, there is little to no functional utility. This requirement renders the kidney the least mature among bioprinting targets, despite considerable scientific interest [22].

**Figure 3:**
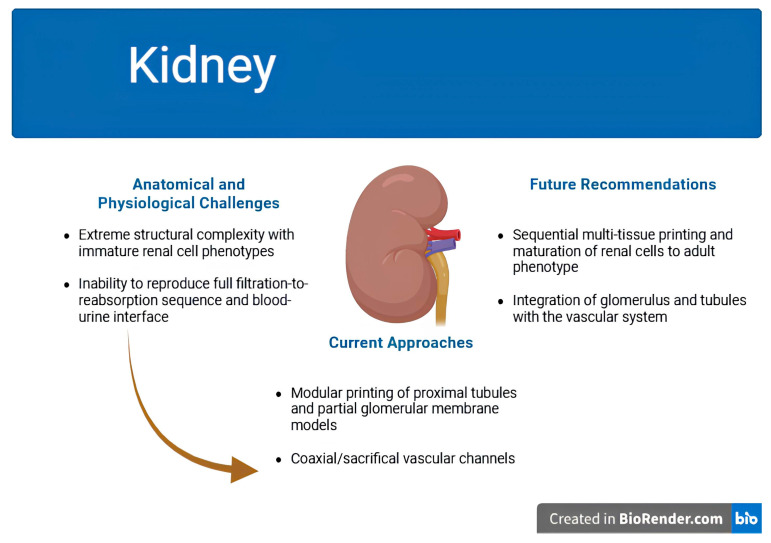
Kidney Bioprinting: Anatomical Complexity, Engineering Strategies, and Research Needs. Created with BioRender.com.

Figure 3 depicts the design of bioprinted kidney constructs, including proximal tubules and glomerulus-mimicking structures. These efforts aim to reproduce filtration, reabsorption, and secretory functions critical to renal physiology.

### 5.2. Modular Bioprinting Strategies and Partial Functional Units

Recognizing the difficulty of printing whole nephrons, researchers have adopted modular strategies focusing on individual components of renal function. The proximal tubule has emerged as the primary target, given its relative structural simplicity, role in nephrotoxicity screening, and potential for vectorial transport modeling. Bioprinted proximal tubule models have been successfully engineered using coaxial and sacrificial printing to generate lumenized epithelial tubes capable of directional glucose, sodium, and protein reabsorption [3,97].

In one representative study, researchers developed perfusable proximal tubule-on-a-chip constructs lined with human proximal tubular epithelial cells that exhibited segment-specific transporter expression, including P-gp, MRP4, and OCT2, and formed tight epithelial barriers [103]. These constructs were functionally challenged with nephrotoxic compounds, such as cisplatin, which induced dose-dependent disruptions in barrier integrity, validating their utility for in vitro nephrotoxicity testing [104]. While these models represent a significant advance for disease modeling and drug screening, they fall short of therapeutic application due to their limited architectural scope, absence of glomerular filtration units, and lack of a vascularized interface.

Attempts to bioprint the glomerular filter, a tripartite structure composed of fenestrated endothelium, a specialized basement membrane, and podocytes, remain largely in the exploratory phase. Some studies have reported partial success in fabricating multilayered membranes with selective permeability to albumin and small molecules, suggesting early steps toward mimicking glomerular function [22]. However, these constructs have not been shown to operate under physiological hydrostatic pressure, nor have they demonstrated sustained filtration linked to downstream tubular processing.

Replicating nephron segments such as the loop of Henle and collecting ducts remains exceptionally challenging due to their intricate spatial architecture and dependence on countercurrent osmotic mechanisms for fluid regulation and urine concentration. As Fransen et al. articulate, bioprinting technology continues to fall short in reproducing such layered, functionally compartmentalized microstructure [105]. Similarly, van den Berg et al. emphasize that successful generation of the branching collecting duct system and extended loop of Henle remains a critical bottleneck in achieving renal functional realism [106].

### 5.3. Cellular Immaturity and Patterning Constraints

Bioprinting renal constructs require not only nephron-specific epithelial cells but also their spatial arrangement into functionally distinct domains. Current protocols for differentiating kidney-relevant cell types from pluripotent stem cells have achieved some success in generating podocytes, proximal tubular cells, and collecting duct-like epithelium, but these cells typically remain immature and exhibit fetal gene expression profiles [22,49]. Moreover, they lack physiological responses to key regulatory hormones such as aldosterone or antidiuretic hormone (ADH), limiting their functional relevance.

Accurate spatial patterning remains another critical bottleneck. A nephron operates as a continuous unit, with seamless transitions between the glomerulus, proximal tubule, loop of Henle, distal tubule, and collecting duct. Yet most bioprinting modalities cannot achieve this level of contiguous multi-tissue fabrication at sub-millimeter resolution. Even within printed proximal tubules, misalignment of epithelial polarity, incomplete basement membrane deposition, and inconsistent lumen formation remain recurring problems [3].

Additionally, supporting cells such as renal interstitial fibroblasts, pericytes, and immune cells, each of which contributes to homeostasis, injury response, and fibrosis, are rarely included in current models. Their exclusion further limits the physiological fidelity of printed tissues and may alter drug response or regenerative potential.

### 5.4. Vascularization and Blood–Urine Interface

No discussion of kidney bioprinting is complete without addressing the need for integrated, bidirectional vascular networks. Unlike many organs, the kidney is both a highly perfused and filtration-centric organ, receiving approximately 20% of the cardiac output, nearly 1 L per minute, and filtering roughly 180 L of plasma per day (about 125 mL/min) [107]. Its microvascular architecture must support both ultrafiltration in the glomerulus and reabsorption/secretion across the peritubular capillaries and vasa recta.

Efforts to replicate this vascular complexity through coaxial printing, endothelialization of microchannels, or sacrificial bioinks have yielded only partial success. While endothelialized channels can sustain perfusion in vitro and prevent central necrosis, they fall short of reproducing the fenestrated endothelium and hemodynamic forces required for active filtration and solute exchange [3,43]. Moreover, no existing platform has demonstrated a functional blood-to-urine interface, an essential feature for removing metabolic waste and regulating fluid homeostasis.

Another concern is the mismatch between printed vascular scaffolds and the mechanical environment of renal tissue. The kidney experiences cyclical pressure changes associated with glomerular filtration and urine flow. Printed constructs must therefore endure dynamic stress while preserving lumen patency, selective permeability, and endothelial stability, requirements that remain unmet in current designs [22].

### 5.5. Realistic Applications and Clinical Translation Pathways

Given the immense complexity of fabricating a whole kidney, initial translational efforts are expected to focus on auxiliary applications. These may include implantable renal assist devices that provide temporary detoxification, printed constructs for nephrotoxicity testing in drug development, or miniaturized nephron units for modeling genetic and acquired renal diseases [22].

The regulatory path for such constructs will likely depend on function-specific endpoints. For filtration, this may involve measurement of creatinine clearance, urea excretion, and electrolyte regulation under controlled perfusion. For secretory or hormonal applications, relevant biomarkers such as erythropoietin or renin levels may serve as functional readouts. Yet most studies to date have focused on structural or histological assessments, which, while valuable, are not sufficient for regulatory approval [12].

Safety concerns, especially immune compatibility and fibrotic encapsulation, also remain underexplored in renal constructs. Osidak et al. and Ricci et al. caution that printed tissues derived from dECM or xenogeneic cells may trigger chronic inflammation, particularly given the kidney’s role in systemic immune modulation [12,49]. Longitudinal preclinical trials in large immunocompetent animals will be essential before human studies can proceed.

## 6. Pancreatic Bioprinting—Balancing Function, Vascularization, and Immune Tolerance

### 6.1. Therapeutic Motivation and Biological Constraints

Among the organs explored for 3D bioprinting, the pancreas, specifically its endocrine compartment, presents a uniquely defined clinical target: the restoration of insulin secretion in patients with type 1 diabetes mellitus (T1DM). Unlike liver or kidney bioprinting, where multiple complex physiological functions must be replicated in parallel, endocrine pancreatic constructs are evaluated primarily by their ability to sense glucose and release insulin in a regulated, timely manner [23].

This functional specificity simplifies the goals of construct design. However, the pancreas also presents a set of tightly coupled challenges. Islets of Langerhans, which constitute the functional endocrine units of the pancreas, are heterogeneous microorgans composed of multiple cell types: insulin-producing β-cells (~60%), glucagon-producing α-cells (~20%), somatostatin-producing δ-cells (~10%), and minor populations of PP and ε-cells. These cells engage in local paracrine signaling to fine-tune hormone release and maintain glucose homeostasis. Any engineered tissue must therefore preserve not only β-cell viability but also the cellular diversity and microarchitecture of the native islet [23,52].

Moreover, endocrine pancreatic function is highly dependent on vascularization. Islets are among the most richly perfused tissues in the human body, with dense capillary networks that allow minute-to-minute sensing of blood glucose and immediate hormone diffusion. Bioprinted constructs that lack perfusion often show delayed or blunted insulin responses, limiting their clinical relevance. Finally, the autoimmune etiology of T1DM adds a layer of complexity: any implanted islet-like construct must also evade immune recognition or destruction, which has driven research into encapsulation, immunomodulation, and scaffold design [24].

**Figure 4:**
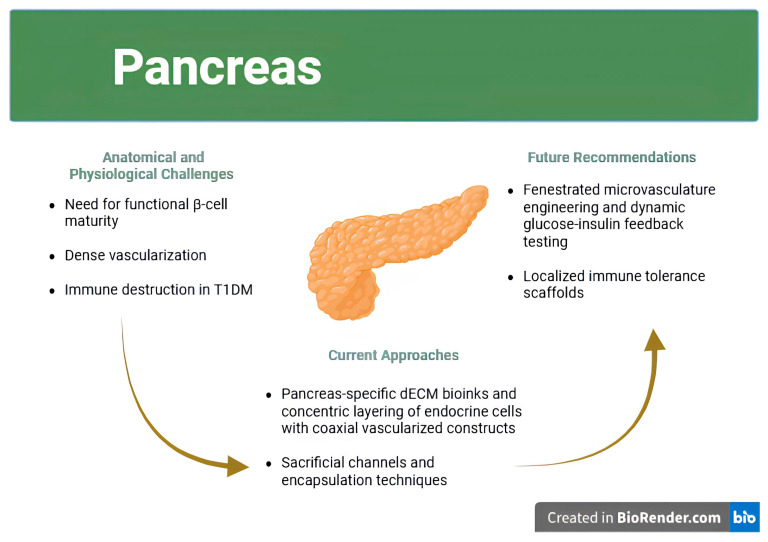
Pancreas Bioprinting: Anatomical Challenges, Current Approaches, and Future Directions. Created with BioRender.com.

As represented in Figure 4, pancreatic bioprinting strategies focus on recreating vascularized islet-like clusters. These constructs support insulin secretion and attempt to integrate with host vasculature while avoiding immune detection.

### 6.2. Cellular Sources and Islet Microarchitecture

Cell sourcing remains one of the most pivotal decisions in pancreatic bioprinting. Native human islets are functionally ideal but are limited by donor availability, inter-donor variability, and reduced viability during in vitro manipulation. Stem cell–derived β-like cells, often differentiated from hiPSCs or hESCs, offer a renewable alternative but generally exhibit immature secretory profiles, including delayed response times, low insulin output, and incomplete expression of key transcription factors such as PDX1 and MAFA [23,52].

Strategies to enhance β-cell maturity include prolonged differentiation protocols, co-culture with endothelial or mesenchymal stromal cells, and use of tissue-specific bioinks such as pancreas-derived dECM. For instance, Ribezzi et al. demonstrated that β-cells printed within pancreas-specific dECM scaffolds showed significantly improved insulin gene expression and glucose-stimulated insulin secretion compared to those cultured in standard GelMA matrices [52]. This highlights the critical role of biochemical cues in guiding endocrine function and underscores the need for tissue-mimetic scaffolds.

Equally important is the preservation of islet microarchitecture. Native islets exhibit a precise spatial distribution of α-, β-, and δ-cells that optimizes hormonal crosstalk. Bioprinting offers a unique advantage in this regard, allowing controlled deposition of each cell type in defined ratios and patterns. Several groups have employed droplet or laser-assisted printing to create pseudo-islets with concentric layering of endocrine cells [108]. These constructs mimic the natural anatomy more closely than random cell aggregates and exhibit improved insulin dynamics and metabolic stability [23]. Multimaterial voxelated printing has further enabled spatially defined deposition of different endocrine cell populations within islet-mimetic architectures, offering improved compositional fidelity and functional compartmentalization [108].

### 6.3. Vascularization and Dynamic Glucose Responsiveness

Among the most technically demanding aspects of pancreatic bioprinting is the recreation of the islet–vascular interface, which is essential for maintaining glucose-sensing and insulin secretion dynamics. Native islets are densely vascularized, with intricate capillary networks that facilitate rapid hormonal exchange. Replicating this architecture in engineered constructs remains a critical barrier to achieving physiologically relevant function. Glucose sensing and insulin release operate on minute-to-minute timescales and require intimate contact between β-cells and perfused microvasculature. In the absence of functional vasculature, bioprinted islets are prone to central necrosis, exhibit delayed insulin kinetics, and demonstrate poor post-implantation survival. A major engineering challenge in pancreatic bioprinting is replicating the islet’s vascular interface, which is critical for nutrient delivery, insulin secretion, and proper integration with host vasculature [23,24].

Coaxial printing has enabled the creation of vascularized hydrogel strands that enclose β-cells in perfused lumens, facilitating oxygenation and nutrient delivery [109]. In some designs, an inner endothelial channel is surrounded by concentric rings of endocrine cells and supporting matrix, forming an artificial “islet–capillary unit.” Sanyal and Mallick showed that such constructs exhibited improved insulin release under glucose challenge and maintained viability longer than non-vascularized counterparts [23].

Another effective strategy uses sacrificial inks, such as Pluronic F127 or gelatin, that are removed post-printing to create perfusable microchannels, which are then lined with endothelial cells to support vascularization. These endothelialized constructs have demonstrated the ability to maintain functional capillary-like networks in vitro and promote spontaneous inosculation with host vasculature upon implantation, a critical step toward long-term viability [110,111]. Despite this progress, engineering a fully functional capillary bed, complete with fenestrated endothelium, pericyte stabilization, and hormonal feedback mechanisms, remains an unmet challenge.

Quantitative validation of dynamic responsiveness is still rare. Most studies report glucose-stimulated insulin secretion (GSIS) at static glucose concentrations rather than real-time assays of pulsatility, latency, and amplitude of insulin release. For constructs to be considered functionally equivalent to native islets, they must demonstrate not only correct hormone output but also rapid return to baseline levels and reproducibility over days to weeks [23].

### 6.4. Immune Protection and Scaffold Design

Immune rejection remains one of the primary causes of failure in pancreatic transplantation. In type 1 diabetes (T1D), autoreactive T-cells infiltrate pancreatic islets, targeting β-cell antigens and leading to progressive islet destruction (*insulitis*) that undermines graft survival [112,113]. As such, bioprinted pancreatic tissues must not only restore functional insulin secretion but also evade or resist immune attack to be therapeutically viable.

Encapsulation technologies have been widely explored, including hydrogel microcapsules, semi-permeable membranes, and immune-modulatory coatings. These approaches aim to block immune cell infiltration while allowing diffusion of glucose and insulin. However, encapsulation often impairs mass transfer, leading to hypoxia, β-cell stress, and apoptosis [24].

Recent scaffold designs have attempted to strike a balance between immunoprotection and function. Ribezzi et al. reported that printing β-cells within multilayered dECM hydrogels reduced pro-inflammatory cytokine release and minimized immune activation in vitro [52]. Others have explored incorporating immunosuppressive agents or FasL-expressing stromal cells directly into the bioink to induce local immune tolerance. While promising, these strategies remain in early preclinical stages and must be validated in immunocompetent animal models before human translation [12].

### 6.5. Translational Considerations and First-in-Human Applications

While pancreatic bioprinting has shown conceptual promise, particularly in the fabrication of vascularized islet-like constructs and immune-protective scaffolds, clinical translation remains firmly preclinical. Notably, Parvaneh et al. demonstrated that incorporating endothelial and supportive cells can enhance vasculogenesis and immune modulation in islet constructs, yet persistent hypoxia within encapsulated cores continues to hinder functional performance [114]. Similarly, Pignatelli et al. argue that recreating a structurally accurate, vascularized peri-islet niche remains essential but technically elusive [115]. Consequently, critical hurdles remain, such as incomplete β-cell maturation, insufficient vascular integration, and inadequate immune protection, that limit the clinical applicability of bioprinted pancreatic tissues.

Bioprinted pancreatic constructs could overcome these barriers by providing scalable, autologous alternatives that are pre-vascularized and potentially immuno-shielded. The first clinical applications are likely to be subcutaneous or omental implants that release insulin in response to glucose, serving as functional replacements for daily injections or insulin pumps. Outcome metrics would include HbA1c levels, insulin independence, and frequency of hypoglycemic events, alongside safety endpoints such as fibrosis, graft loss, or systemic immune activation [12,24].

## 7. Regulatory Landscape and Clinical Translation—Bridging Innovation and Implementation

### 7.1. Bioprinted Constructs and the Regulatory Grey Zone

Bioprinted organs and tissues defy traditional medical product classifications. They combine living cells, typically regulated as biologics, scaffolding materials, regulated as devices, and occasionally drug-eluting components. Consequently, most regulatory bodies treat bioprinted constructs as combination products, triggering multifaceted review pathways and jurisdictional ambiguities [12,116]. For example, in the United States, the Food and Drug Administration (FDA) splits oversight among the Center for Biologics Evaluation and Research (CBER), the Center for Devices and Radiological Health (CDRH), and the Center for Drug Evaluation and Research (CDER). This fragmentation has delayed the establishment of cohesive guidance specific to bioprinting.

For both manufacturers and regulators of products that merge elements or exhibit characteristics from multiple categories, the classification process has become increasingly complicated. It is essential to grasp how these combination products align with the existing regulatory frameworks from the very beginning of development to fully realize the potential clinical benefits of these technologies [117].

To address the growing complexity of advanced therapies, the FDA created the Regenerative Medicine Advanced Therapy (RMAT) designation, allowing accelerated review for qualified cell- and tissue-based products. However, few bioprinted products have reached the threshold of clinical readiness necessary to apply. In the European Union, the European Medicines Agency (EMA) evaluates bioprinted constructs as Advanced Therapy Medicinal Products (ATMPs) through the Committee for Advanced Therapies (CAT). This framework centralizes the regulation of gene and cell therapies, but country-level discretion, such as hospital exemptions or compassionate use allowances, continues to cause fragmentation across member states [12].

Interestingly, countries such as China and South Korea have adopted more agile frameworks to encourage innovation. China’s National Medical Products Administration (NMPA) has created regenerative medicine pilot zones and allowed conditional clinical use of certain 3D-printed products, including cartilage implants. While this has catalyzed domestic innovation, critics note that global harmonization remains lacking, and concerns persist regarding the long-term safety and standardization of rapidly approved devices [12,116,118].

### 7.2. Functional Uncertainty and Lack of Standardized Potency Metrics

A persistent obstacle to the regulatory approval of bioprinted constructs is the absence of validated, standardized potency assays. Unlike pharmaceuticals, where Critical Quality Attributes (CQAs) are well-defined and routinely applied, bioprinted tissues are complex, dynamic, and bespoke, making consistent potency evaluation difficult. Mladenovska et al. highlight the regulatory challenges unique to 3D bioprinting, including the fragmentation of regulatory pathways, lack of standardization, and unclear quality control frameworks, which collectively hinder clinical translation [119]. In contrast to small-molecule drugs, where purity and pharmacokinetics can be rigorously characterized, a bioprinted liver patch cannot be assessed solely on albumin output; it must also demonstrate sustained enzyme function, bilirubin clearance, and durable integration with host vasculature in vivo [11,21]. These complexities underscore the urgent need for construct-specific, consensus-driven CQAs to guide regulatory approval and clinical deployment of biofabricated tissues.

The same principle applies to cardiac, renal, and pancreatic constructs. Without agreed-upon endpoints and performance thresholds, regulators cannot effectively compare products or make risk–benefit assessments. Ricci et al. emphasized the importance of CQAs, measurable properties such as cell viability, scaffold integrity, vascular perfusability, and hormonal responsiveness, that can predict in vivo functionality and clinical performance [12]. However, the field currently lacks consensus on what CQAs are most relevant for different organ types, and even fewer are validated in animal models.

Another regulatory challenge is batch-to-batch consistency [120]. Most bioprinted constructs are produced under laboratory-scale conditions with high variability in printer settings, bioink composition, cell source, and crosslinking parameters. For widespread clinical use, developers must transition to Good Manufacturing Practice (GMP) conditions, which require validated protocols, sterile production, and rigorous release testing. These infrastructure and compliance demands represent a steep barrier for academic labs and small biotech companies [12].

3D bioprinted organs submitted for clinical transformation approval must be produced in facilities adhering to Good Manufacturing Practices (GMP), utilizing clinical-grade raw materials that have clearly defined Quality Attributes (QAs) [121,122]. Additionally, these items must undergo clinical testing in accordance with Good Clinical Practice (GCP) standards [120].

In addition, achieving complete characterization is likely unattainable, necessitating more comprehensive preclinical and in vivo safety evaluations, along with in-process controls, to adequately assess the risk versus benefit [117,119].

This issue is especially difficult because existing methods for thorough biological and biomechanical characterization are often destructive, compromising the very samples that require analysis; however, some non-destructive techniques are being developed [119,123].

### 7.3. Designing Ethical and Feasible Clinical Trials

Since 2016, only 11 trials related to bioprinting have been recorded, which is minimal compared to the more than 3000 studies on bioprinting published since 2007. This might indicate the overall challenges of bringing tissue engineering into clinical practice [124].

The majority of trials were carried out by teams in China, with the United States and South Korea following closely, which makes these nations the leading contributors in terms of publishing and funding research in bioprinting overall [125,126].

Designing clinical trials for bioprinted tissues introduces ethical and logistical complexities. Many target conditions, such as end-stage organ failure, are life-threatening and lack effective therapies. This creates both urgency and risk. Regulators will likely require initial trials to begin with adjunctive applications such as cardiac patches to support infarcted myocardium or insulin-releasing implants for brittle diabetes, rather than full organ replacements. These constructs can be tested in parallel with standard-of-care therapies, reducing risk to participants [12,24].

Moreover, traditional trial endpoints such as overall survival may be insufficient or inappropriate. For example, hepatic constructs could be evaluated using biochemical markers, while cardiac patches might use echocardiographic ejection fraction or MRI-based infarct size reduction. In pancreatic constructs, time to insulin independence, glycemic control (HbA1c), and insulin secretion kinetics may serve as primary endpoints. Adaptive trial designs, such as single-arm feasibility studies, biomarker-driven endpoints, or historical control comparisons, may offer pragmatic pathways to early-phase validation [12,116].

Importantly, these trials must include long-term follow-up. The risk of chronic inflammation, fibrosis, ectopic tissue formation, or oncogenic transformation, particularly with stem cell–derived constructs, cannot be ruled out by 30- or 60-day endpoints. Regulators are likely to mandate at least 12–24 months of follow-up, including imaging, immunologic profiling, and possible tissue biopsies [12].

## 8. Conclusions

Bioprinting has progressed from a conceptual innovation to a powerful platform with the potential to revolutionize regenerative medicine. Yet, the field stands at a critical inflection point. Future success will not be defined by the sophistication of constructs fabricated in the laboratory, but rather by their durability, integration, and functional reliability once implanted in patients. This review aimed to shift the focus from structural replication to functional performance, advocating for a “function-first” lens that prioritizes electromechanical fidelity, metabolic activity, and therapeutic efficacy over mere architectural fidelity.

To this end, we emphasize the need for standardized, organ-specific potency assays and translationally relevant benchmarks such as ejection fraction, enzyme kinetics, and insulin–glucose dynamics. The heterogeneity of tissue constructs, combined with variability in biomaterials and fabrication methods, underscores the urgency for international consensus on clinical-grade criteria and construct-specific CQAs. Without such benchmarks, the regulatory path to clinical approval remains fragmented and uncertain.

Moreover, this review highlights the pressing research and knowledge gaps across organ systems, from immature hiPSC-derived phenotypes in cardiac tissues, to inadequate capillary-level perfusion in liver and pancreatic constructs, and the absence of functional nephron units in renal bioprinting. Addressing these challenges will require interdisciplinary collaboration among bioengineers, cell biologists, and clinicians, as well as robust preclinical validation in large-animal models. While the hurdles are significant, the translational potential of bioprinting is equally compelling. Moving forward, the integration of AI-guided design, immuno-tolerant biomaterials, and personalized bioinks may define the next frontier. Ultimately, bridging the gap between fabrication and function will determine whether bioprinting evolves from experimental technology to a viable clinical solution.

## Figures and Tables

**Table 1:** Summary of Organ Complexity and Bioprinting Challenges.

Organ	Anatomical Complexity	Physiological Functions	Key Challenges in Bioprinting
Heart	High	Contraction, conduction	Synchronized electromechanical activity, vascularization, immune response, electrical integration [20]
Liver	Moderate–High	Metabolism, detoxification	Lobular zonation, sinusoidal vasculature, long-term viability, fibrosis [11,21]
Kidney	Very High	Filtration, excretion	Nephron reconstruction, segment-specific function, vascular–epithelial interface [22]
Pancreas	Moderate	Endocrine hormone secretion	Vascularization, immune evasion, β-cell maturity and glucose responsiveness [23,24]

**Table 2:** Bioprinting Techniques: Technical Characteristics and Applications.

Modality	Resolution	Bioink Viscosity Range	Cell Viability	Strengths	Limitations
Extrusion	100–300 µm	Medium to high	Moderate (~60–80%)	High cell density, bulk construct formation [3,43]	Low resolution, shear-induced damage
Inkjet	50–100 µm	Low	High (~85–95%)	High spatial precision, suitable for patterning [3,7,8,43]	Limited to low-viscosity inks, nozzle clogging
Laser-Assisted (LAB)	20–100 µm	Low to medium	High (>90%)	Nozzle-free, high precision [3,43]	Expensive, difficult to scale up
SLA/DLP	<50 µm	Low	Moderate to high	Excellent for complex microarchitectures [3,7,8,43]	Limited to photocrosslinkable inks, light-induced cytotoxicity

**Table 3:** Organ-Specific Challenges, Current Strategies, and Future Directions in Bioprinting of Complex Tissues.

Organ	Key Challenge	Current Approaches Mentioned	Remaining Gaps	Future Directions Suggested	References
Heart	Immature hiPSC-derived cardiomyocytes Lack of synchronized electromechanical integration Limited vascularization Mechanical mismatch with host myocardium	Coaxial extrusion for perfusable lumens Alignment-controlled printing to mimic anisotropy Conductive materials (e,g., Gold nanowares) for maturation FRESH- printed ventricular models	Immature electrophysiology and contractility Poor long-term vascular integration Limited large-animal/long-term data	Enhance cardiomyocytes maturity Develop vascular anastomosis methods Standardize functional assessment (ejection fraction, conduction mapping)	[73,74,75,76,77,78]
Liver	Difficulty recreating metabolic zonation Inadequate vascularization; short-term viability Immune rejection and fibrosis	Co-culture with non-parenchymal cell Liver-specific dECM bioinks Coaxial and sacrificial printing for channels FRESH for complex lobule geometry	Failure to achieve oxygen/nutrient gradients Insufficient capillary resolution (<20 μm) Limited long-term immune compatibility studies	Spatially patterned perfusion for zonation Microcapillary-scale printing Immuno-compatible biomaterials	[73,78,79,80,81,82]
Kidney	Extreme structural complexity (nephron-level integration) Immature renal cell phenotypes Inability to reproduce full filtration-to-reabsorption sequence Lack of blood–urine interface	Modular printing of proximal tubules Partial glomerular membrane models Coaxial/sacrificial vascular channels	No functional nephron with continuous segments Lack of hormonal responsiveness Poor vascularization matching renal pressures	Sequential multi-tissue printing at sub-millimeter resolution Maturation of renal cells to adult phenotype Integrate glomerulus and tubules with vascular system	[78,83,84,85,86,87]
Pancreas	Need for functional β-cell maturity Dense vascularization; immune destruction in T1DM	Pancreas-specific dECM bioinks Concentric layering of endocrine cell types; coaxial vascularized constructs Sacrificial channels Encapsulation techniques	Incomplete capillary bed replication Delayed insulin response Immune evasion strategies not clinically validated	Fenestrated microvasculature engineering Dynamic glucose–insulin feedback testing Localized immune tolerance scaffolds	[23,88,89]

**Table 4:** Common Bioink Formulations in Organ Bioprinting.

Bioink Composition	Type	Applications	Advantages	Limitations	References
Alginate	Natural	Cartilage, liver, skin	Biocompatible, easy gelation	Poor cell adhesion, low mechanical strength	[48,57]
Gelatin Methacryloyl (GelMA)	Natural/Hydrogel	Cardiac, cartilage, vascular	Cell-friendly, photocrosslinkable	Weak structural stability alone	[4,48]
Polycaprolactone (PCL)	Synthetic	Bone, vascular scaffolds	Strong, slow degrading, thermoplastic	Bioinert; requires functionalization	[58,61,90]
PEGDA	Synthetic	Liver, vascular tissues	Tunable stiffness, photopolymerizable	Poor bioactivity unless functionalized	[4,62,91]
Alginate–Nanocellulose–Gelatin	Hybrid	Meniscus, soft tissues	High print fidelity, cell viability	Requires fine rheological tuning	[66]
Pancreas-specific dECM	Natural/dECM	Pancreatic islet maturation	Tissue-specific cues, enhanced insulin expression	Batch variability, regulatory risk	[23,52]
FRESH-based Gelatin Support Printing	Process-based	Cardiac, vascularized soft tissues	Enables complex geometry, maintains structure	Labor-intensive, challenging scale-up	[20,48]

## Data Availability

No new data were created or analyzed in this study. Data sharing is not applicable to this article.
